# Influence of combined abiotic/biotic factors on decay of *P. aeruginosa* and *E. coli* in Rhine River water

**DOI:** 10.1007/s00253-024-13128-z

**Published:** 2024-04-10

**Authors:** Sha Gao, Nora B. Sutton, Thomas V. Wagner, Huub H. M. Rijnaarts, Paul W. J. J. van der Wielen

**Affiliations:** 1 Department of Environmental Technology, Wageningen University, PO Box 17, 6700EV Wageningen, The Netherlands; 2 KWR Water Research Institute, Groningenhaven 7, 3433PE, Nieuwegein, The Netherlands; 3 Laboratory of Microbiology, Wageningen University, PO Box 17, 6700EV Wageningen, The Netherlands

**Keywords:** Fecal indicator bacteria, Opportunistic pathogen, Microbiological surface water quality, Interactive effect

## Abstract

**Abstract:**

Understanding the dynamic change in abundance of both fecal and opportunistic waterborne pathogens in urban surface water under different abiotic and biotic factors helps the prediction of microbiological water quality and protection of public health during recreational activities, such as swimming. However, a comprehensive understanding of the interaction among various factors on pathogen behavior in surface water is missing. In this study, the effect of salinity, light, and temperature and the presence of indigenous microbiota, on the decay/persistence of *Escherichia coli* and *Pseudomonas aeruginosa* in Rhine River water were tested during 7 days of incubation with varying salinity (0.4, 5.4, 9.4, and 15.4 ppt), with light under a light/dark regime (light/dark) and without light (dark), temperature (3, 12, and 20 °C), and presence/absence of indigenous microbiota. The results demonstrated that light, indigenous microbiota, and temperature significantly impacted the decay of *E. coli*. Moreover, a significant (*p*<0.01) four-factor interactive impact of these four environmental conditions on *E. coli* decay was observed. However, for *P. aeruginosa*, temperature and indigenous microbiota were two determinate factors on the decay or growth. A significant three-factor interactive impact between indigenous microbiota, temperature, and salinity (*p*<0.01); indigenous microbiota, light, and temperature (*p*<0.01); and light, temperature, and salinity (*p*<0.05) on the decay of *P. aeruginosa* was found. Due to these interactive effects, caution should be taken when predicting decay/persistence of *E. coli* and *P. aeruginosa* in surface water based on a single environmental condition. In addition, the different response of *E. coli* and *P. aeruginosa* to the environmental conditions highlights that *E. coli* monitoring alone underestimates health risks of surface water by non-fecal opportunistic pathogens, such as *P. aeruginosa*.

**Key points:**

- *Abiotic and biotic factors interactively affect decay of E. coli and P. aeruginosa*
- *E.coli and P.aeruginosa behave significantly different under the given conditions*
- *Only E. coli as an indicator underestimates the microbiological water quality*

**Supplementary Information:**

The online version contains supplementary material available at 10.1007/s00253-024-13128-z.

## Introduction

Amsterdam is the capital city of the Netherlands and is known for its extensive waterways in the form of canals connected to the Amstel River (a tributary of the Rhine River) which serve as transportation links and recreation venues that also support tourism. Especially the innercity canals are more often used for swimming events and water sports (Hintaran et al. 2018). Although the local government favors swimming in the canals, only nine official swimming locations are assigned to five lakes and four canals that are also monitored regularly according to European Union bathing water regulations (Peters et al. 2021). In contrast, there are over 40 other unofficial swimming sites that are not monitored or regulated. Health issues have been reported in the past during and after several organized swimming events in the inner canals of Amsterdam. For example, after the Amsterdam City Swim in 2015, 31% of the contestants reported to suffer from gastroenteritis after the event (Hintaran et al. 2018). In addition to Amsterdam, many other cities worldwide are confronted with an increasing (often non-regulated) use of urban waters for swimming (Van Der Meulen et al. 2023). More insights in the cause of these swimming-related health problems and potential measures to mitigate these are, therefore, needed to protect public health during recreational use of urban water.

Skin complaints and gastrointestinal illness are reported as the most commonly identified health problems after swimming in open waters (Schets et al. 2008). A cause for the abovementioned health complaints could be the presence of fecal and opportunistic waterborne pathogens in the surface water. Fecal pathogens, such as *Norovirus*, *Salmonella*, and *Campylobacter*, enter into surface water via discharged wastewater treatment plant effluent, rainwater runoff, sewage overflow, waterfowl, and other birds dropping, etc. (Aw 2019). Adapted to host intestines, upon entry into the aquatic environment, fecal pathogens are often subjected to decay instead of growth because of the different environmental conditions (Korajkic et al. 2019b). Hence, the microbial water quality can be restored within a certain time (days) after an incident input such as a heavy rainfall event with the emission of fecal bacteria into the water body (Sales-Ortells et al. 2015). Fecal indicator bacteria (FIB), such as intestinal enterococci and *Escherichia coli*, are generally used to indicate the level of fecal contamination of surface water according to the WHO and European Union bathing water regulations (World Health Organization 2003; E. U. Directive 2006). Most studies have shown that fecal pathogens decay in water bodies (Korajkic et al. 2019a, 2019b; Dean and Mitchell 2022). However, certain other waterborne pathogens, such as *Pseudomonas aeruginosa* and *Vibrio cholerae*, can persist or even grow/reproduce in the surface water environment (Aw 2019). Hence, these organisms may pollute water, after emission into the water, for much longer time frames. Indicator organisms for these opportunistic pathogens able to grow in the aquatic environment have not been identified and, consequently, potential health risks of swimming in surface water that relate to such organisms can be underestimated or even overlooked (Januário et al. 2019). For instance, between 1991 and 2007 in the Netherlands, there were 17 ear infection outbreaks from recreational swimming, of which 16 were attributed to *P. aeruginosa*, which is one of the most versatile opportunistic pathogens (Schets et al. 2011). Furthermore, a study showed that *P. aeruginosa* was one of the waterborne pathogens that were responsible for most hospitalizations and deaths in the USA (Collier et al. 2021).

Extensive research has been conducted on the decay/persistence behavior of mainly fecal pathogens in surface water at different abiotic and biotic conditions (Noble et al. 2004; Jenkins et al. 2011; Scoullos et al. 2019; Korajkic et al. 2019b). Among the abiotic factors, sunlight, temperature, and salinity have emerged as crucial determinants for decay/persistence (Sinton et al. 1994; Whitman et al. 2004; Ibrahim et al. 2019; Dean and Mitchell 2022). Sunlight, particularly its UV component, inhibits bacterial survival by damaging DNA through mutations and generating photo-oxidative radicals (Nelson et al. 2018). Since UV is fully absorbed by a 40-cm water column, depth of the water body is also of importance (He et al. 2016). At temperature lower than 10–15°C, it has been observed that inactivation of both FIB and pathogenic fecal pathogens is lower than at higher temperatures (>15°C) (Sinton et al. 1994; Atlas and Bartha 1998; Sokolova et al. 2012; Korajkic et al. 2019b). In Amsterdam, the urban waters have generally a depth of more than 1.5 m, and temperatures fluctuate between close to 0 (winter) and 25 °C (summer) (Van Der Meulen et al. 2023). Salinity is also a factor since the Amsterdam canal network ends in main waterways connected to the open sea via ship-locks. Research shows that higher salinity causes higher inactivation for FIB (Liang et al. 2017; Korajkic et al. 2018). Additionally, the survival or growth of fecal and non-fecal waterborne pathogenic bacteria is also influenced by various biotic processes, including predation by flagellated and ciliated protozoa and competition for nutrients with other bacteria (Korajkic et al. 2019b). For instance, predation alone has shown to cause up to 90% of both fecal and indigenous bacterial mortality (Rodríguez-Zaragoza 1994; Menon et al. 2003).

Previous studies investigated the effect of different environmental conditions on the decay/persistence of FIB and other waterborne pathogens. For example, sunlight exposure has been identified as a primary individual inhibitor of FIB survival compared to temperature, predation, and salinities in different studies (Nelson et al. 2018; Ibrahim et al. 2019; Dean and Mitchell 2022). For *E. coli*, the decay could be explained by competition with or even predation by the indigenous microbiota (49.2%) versus salinity (40.1%) as tested in outdoor fresh versus marine water mesocosms (Korajkic et al. 2013). The same research group, however, concluded from additional experiments in mesocosms that salinity appeared to be the most influential factor affecting the decay rate of FIB (Korajkic et al. 2019a). The lack of studies on opportunistic waterborne pathogens like *P. aeruginosa* makes it difficult to determine the persistence of these bacteria in surface water under different environmental conditions. Studies have been published showing that the growth of *P. aeruginosa* was temperature dependent and that *P. aeruginosa* was able to grow at 15°C with increasing growth rate and biofilm formation at higher temperatures (30°C) (Schets et al. 2020; Van Der Wielen et al. 2023). In addition to temperature dependence, *P. aeruginosa* was reported to survive in salinity levels that ranges from 0 to 7 ppt in artificial marine water regardless of temperature changes (Khan et al. 2010). Moreover, *P. aeruginosa* was reported to be more resistant to UV exposure than other bacteria (Mena and Gerba 2009; Wnlfe 1990).

Only a few studies investigated the potential interactive effects of environmental conditions on the persistence of waterborne pathogens and FIB in surface water (Liang et al. 2017; Dean and Mitchell 2022). These studies only investigated the interactive effects of two different environmental conditions and the results suggested that interactive effects can occur, but whether more than two conditions exhibits interactive effects as well remains unknown. The main objective of our research is to study the influence of individual and combined abiotic (temperature, salinity, and sunlight) and biotic (indigenous microbiota) environmental conditions on the decay/persistence of *E. coli* and *P. aeruginosa* in Rhine River water that feeds the urban Amsterdam water system. In addition, a comparison of the behavior of these two bacteria was conducted to determine whether *E. coli* is a reliable indicator organism for the fate of opportunistic waterborne pathogens.

## Materials and methods

### Inoculum and Rhine River water

#### Rhine River water

Ten L Rhine River water was taken near the city Wageningen in the Netherlands. River water was sampled 10–20 cm below the surface of the river and immediately transported to the laboratory at 4°C. The river water samples taken between October 2021 and February 2022 were used for studying decay of *E. coli*. The river water samples for the experiments with *P. aeruginosa* were taken during May 2022 to August 2022. After transportation to the laboratory, all water samples were stored at 4°C and used within 48 h. The temperature of the sampled river water was measured in the field by a temperature meter. The electrical conductivity (EC), pH, ammonium (NH_4_^+^-N), and chemical oxygen demand (COD) of the river water samples were tested after the water had been transported to the laboratory (Table S1, Table S2).

#### Inoculum preparation

*E. coli* DSM1103 and *P. aeruginosa* DSM939 were both obtained from DSMZ-German Collection of Microorganisms and Cell Cultures GmbH (Braunschweig, Germany). *E. coli* DSM1103 was selected as the fecal indicator bacteria in this study as it has been widely studied in environmental-related researchers and it can be easily grown in the laboratory. *P. aeruginosa* DSM939 was chosen as it is one of the standard strains stored in available culture collection and it was isolated from a water source which minimized the influence of matrix on the behavior of bacteria in this study. Frozen dried pure cultures of *E. coli* or *P. aeruginosa* were rehydrated with 1 mL tryptic soy broth (NutriSelect® Basic of Merck KGaA 22092, Darmstadt, Germany), streaked onto tryptic soy agar plates (15g agar in 1000mL tryptic soy broth), and incubated overnight at 37°C. One separate colony of *E. coli* or *P. aeruginosa* was then loop-inoculated into 300 mL M9 medium diluted 5 times from the 5× M9 Minimal Salts (M9956 of Sigma-Aldrich, Merck, Germany; pH 6.6–7.0) with 10 mg/L glucose in 500-mL serum bottle. Each culture was incubated at 35°C and a growth curve was made (Figure S2a and S2b). At the start of the stationary phase, bacteria were collected by centrifuging at the suspension for 10 min at 9000×*g*, washed three times with sterile 0.9% saline, and resuspended in 2.5 mL 0.9% saline water (9 g NaCl in 1L MilliQ water, sterile). This solution was stored at 4°C and used for inoculation for the decay experiments next day.

### Experimental setup

Sterile serum bottles (500mL) were used as experimental microcosms, inoculated with *E. coli* or *P. aeruginosa* and exposed to a mixture of different abiotic factors (light exposure, temperature, and salinity) and a biotic factor (indigenous microbiota) as shown in Table 1.

**Table 1:** Environmental conditions given in this study

Salinity	Light		Temperature	Indigenous microbiota	
0.4ppt	Yes	No	3, 12, 20°C	Yes	No
5.4ppt	Yes	No	3, 12, 20°C	Yes	No
9.4ppt	Yes	No	3, 12, 20°C	Yes	No
15.4ppt*	Yes*	No*	3, 12, 20°C*	Yes*	No*

*Represent conditions only for *P. aeruginosa* but not for *E. coli*

The sterile serum bottles contained 300 mL Rhine River water. Half of the bottles had unfiltered river Rhine River water containing indigenous microbiota and the other half had Rhine River water that was first filtered over a 0.45-μm sterile membrane filter (Whatman, Cytiva, Germany) and subsequently over a 0.2-μm membrane filter (Whatman, Cytiva, Germany) to remove the indigenous microbiota. Part of the bottles were incubated under dark conditions by covering them with aluminum foil, and the other part of the bottles were exposed to a light/dark regime (daily regime of 7 h exposure to 30 μmol m^−2^ s^−1^ using a Xenon lamp followed by 17 h darkness) in a climate chamber. The spectrum of the Xenon lamp was previously described (Wagner et al. 2020). For *E. coli*, the water in one third of the bottles received 1.5 g sea salts (S9883 from Merck, Darmstadt, Germany) (resulting in a salinity of 5.4 ppt), another one third 2.7 g sea salts (resulting a salinity of 9.4 ppt), and the last one third did not receive extra sea salts (salinity of 0.4 ppt, the initial salinity of the Rhine River water). For *P. aeruginosa*, these three salinities, together with a salinity of 15.4 ppt (obtained by adding 4.5 g sea salts to the Rhine River water), were tested. Bottles were incubated at 3, 12, or 20°C for 7 days in a climate chamber, except the bottles with *P. aeruginosa* that were incubated at 12°C in the dark. Those were incubated for 38 days, to determine the persistence of *P. aeruginosa* over a longer time span. This setup resulted in 72 bottles for *E. coli* and 96 bottles for *P. aeruginosa*.

*E. coli* was added to the 72 bottles to obtain a final concentration of 1×10^6^ CFU·mL^−1^ and *P. aeruginosa* was added to the other 96 bottles to obtain the same final concentration. One milliliter of water was sampled from each bottle using a sterile syringe with sterile needle on days 0, 1, 2, 4, and 7. Ten times dilution series of the samples were made with sterile 0.9% saline water and immediately filtered through a sterile 0.45-μm membrane (Whatman, Cytiva, Germany) and placed on a selective agar media for enumeration of *E. coli* or *P. aeruginosa*. The plates has been carefully checked to make sure no air bubbles existed between the membrane and the agar media. If the number of colony-forming units (CFU) for *E. coli* or *P. aeruginosa* from a bottle was below the detection limit before day 7, samples from that bottle were no longer taken.

### Analysis methods

#### Physicochemical parameters

Conductivity and pH were measured with a multi-digital meter (HACH HQ440d, Germany). NH_4_^+^-N and COD were analyzed with HACH Lange GMBH kits and measured with the DR 3900 spectrophotometer (HACH LCK 304 and LCK1414, Germany) (Table S1, Table S2) (Saha et al. 2020). The electrical conductivity of fresh Rhine River water samples was then calculated into salinity by a standard curve (Figure S1). These calculations demonstrated that the Rhine River water samples had an average salinity of 0.4 ± 0.1 ppt.

#### Microbiological parameters

*E. coli* was enumerated on modified Membrane-Thermotolerant *Escherichia coli* (mTEC) agar media (Merck, Germany) following the USEPA Method 1603 (USEPA 2014). Two appropriate dilutions were immediately filtered in duplicate on a 0.45-μm membrane (Whatman, Cytiva, Germany) and the membrane was subsequently incubated on top of the mTEC agar. All plates were incubated at 35°C ± 0.5°C for 2 ± 0.5 h and transferred to Whirl-Pak bags (Merck, Darmstadt, Germany) that were then submerged in a 44.5°C ± 0.2°C water bath for 22 ± 2 h. After incubation, the CFU of *E. coli* were determined by counting the *E. coli* typical purple colonies.

*P. aeruginosa* was counted on *Pseudomonas* CN agar (Merck, Darmstadt, Germany) using the membrane filtration method according to ISO 16266. Two appropriate dilutions were filtered over a 0.45-μm membrane and the membrane was subsequently placed on *Pseudomonas* CN agar. All the plates were then incubated at 36 °C for 44 h. After incubation, the plates were exposed to 360-nm UV light (Merck, Darmstadt, Germany) and the fluorescent colonies were counted, as these are typical for *P. aeruginosa*.

No *E. coli* and *P. aeruginosa* colonies grew on the plates with the corresponding selective agar media when 1 mL of Rhine River water sample was tested. No colonies of *E. coli* or *P. aeruginosa* were obtained when 0.2-μm filtered Rhine River water was tested with the corresponding selective agar media. In addition, heterotrophic plate counts on tryptic soy agar were below 2 CFU·mL^−1^ in 0.2-μm filtered Rhine River water.

### Data analysis

The log-based first-order exponential decay “Chick–Watson” model (Chick 1908) (Eq. (1)) was used to describe the decay/growth of *E. coli* and *P. aeruginosa* in all microcosms. To calculate the decay rate, a linear regression between the logarithm of *C*_*t*_/*C*_0_ values and the incubation time points was performed. The slope of this linear regression line was used to express the decay rate. If the limit of detection was reached before day 7, further time points were not taken into consideration.

1 $${\log}_{10}{~}^{{C}_t}\!\left/ \!{~}_{{C}_0}\right.=- kt$$

where *t* is the time (day), *C*_*t*_ is the concentration of pathogens at time *t*, *C*_0_ is the initial concentration of pathogen at time 0, and *k* (day^−1^) is the first-order decay rate constant.

All statistical analyses were done with the calculated first-order decay rates using IBM SPSS Statistics (version 28.0.0.0). Based on the outcome of the Levene’s test of equality of error variances, decay rates at different temperatures or different salinities were analyzed using a one-way ANOVA with Bonferroni post hoc test or a Kruskal-Wallis *H* test with a Bonferroni post hoc test. Differences in decay rates between light/dark versus dark regime or between presence and absence of indigenous microbiota were analyzed using a one-way ANOVA. The interactive impact between multiple conditions on the decay of *E. coli* or *P. aeruginosa* was examined by performing a four-way ANOVA. Finally, a Student *t*-test was performed with the decay rates of *E. coli* and *P. aeruginosa* under all given conditions to investigate whether the behavior of these two bacteria were significantly different from each other.

## Results

### Decay of *E. coli* in Rhine River water at different conditions

**Fig. 1:**
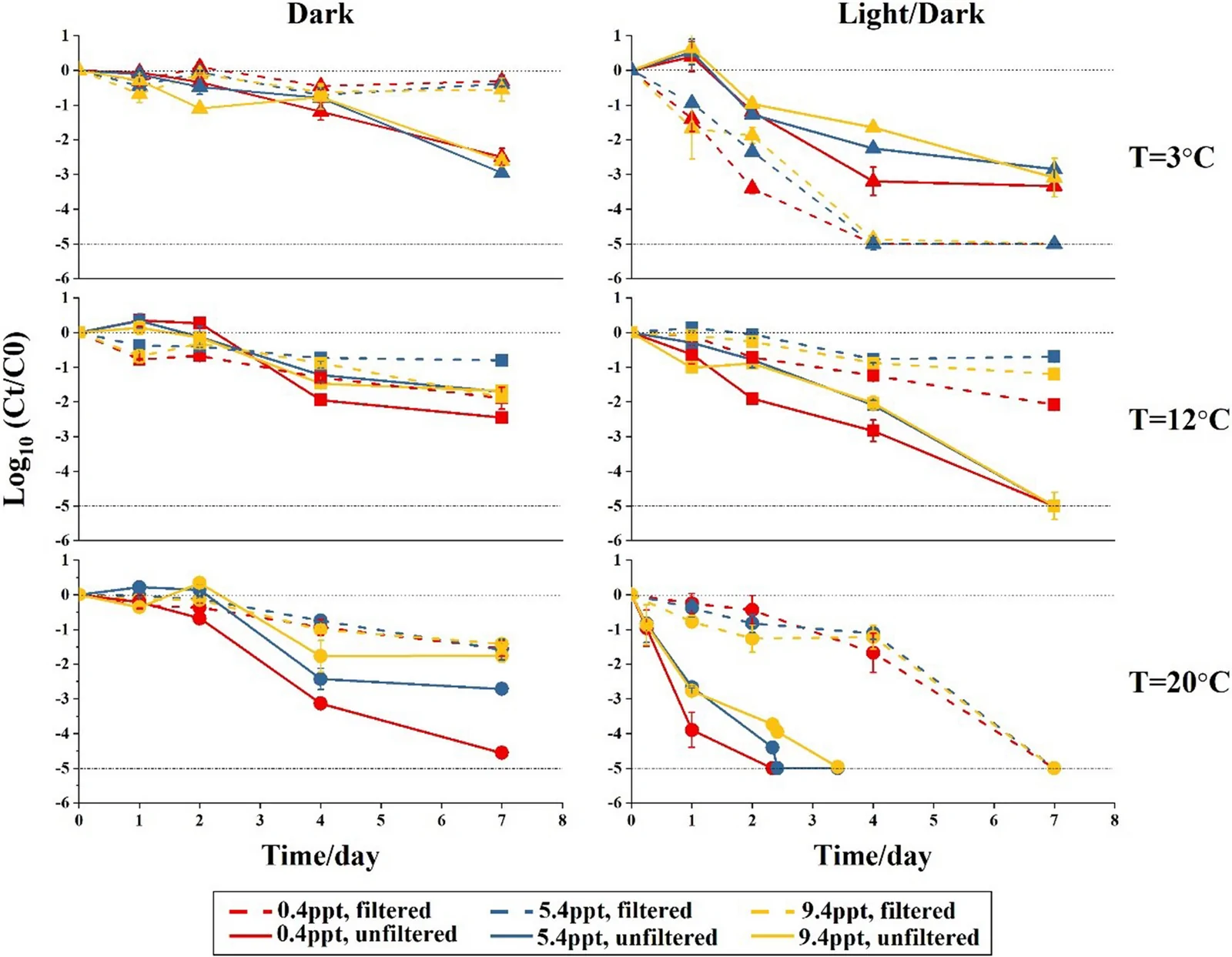
Log_10_ (*C*_*t*_/*C*_0_) of *E. coli* in filtered and unfiltered Rhine River water that were incubated at various salinity and temperature levels under a dark or light/dark regime. Data are averages from duplicate bottles (*n*=2); error bars represent standard deviations. The upper dashed line indicates *C*_*t*_=*C*_0_; the bottom dashed line indicates the detection limit in our study

The decay of *E. coli* in filtered water was found to be less pronounced at the lowest temperature of 3°C compared to the highest temperature of 20°C, at all tested salinities under dark conditions (Fig. 1). Consequently, at 3°C under dark conditions, the first-order decay rates were the lowest among the three temperatures tested (Table 2), and this difference was also statistically significant (*p*<0.05, Table S5). However, this is not the case under light/dark regime where the fastest decay has been observed at 3°C and the slowest decay at 12°C with all salinities (Fig. 1).

**Table 2:** Log_10_-based decay rates of *E. coli* in Rhine River water incubated for 7 days at different environmental conditions in microcosms. Data are averages from duplicate bottles (*n*=2); errors (±) represent standard deviations

Log_10_-based decay rate *E. coli* (d^-1^)				
	3°C- Dark		3°C- Light/Dark	
	Filtered	Unfiltered	Filtered	Unfiltered
0.4 ppt	0.04 ± 0.01	0.36 ± 0.03	1.26 ± 0.03	0.58 ± 0.00
5.4 ppt	0.05 ± 0.01	0.41 ± 0.01	1.25 ± 0.02	0.46 ± 0.01
9.4 ppt	0.06 ± 0.04	0.35 ± 0.00	1.16 ± 0.05	0.50 ± 0.09
	12°C- Dark		12°C- Light/Dark	
	Filtered	Unfiltered	Filtered	Unfiltered
0.4 ppt	0.25 ± 0.05	0.43 ± 0.00	0.31 ± 0.00	0.72 ± 0.05
5.4 ppt	0.13 ± 0.02	0.36 ± 0.07	0.15 ± 0.01	0.72 ± 0.01
9.4 ppt	0.22 ± 0.03	0.33 ± 0.03	0.18 ± 0.03	0.70 ± 0.04
	20°C- Dark		20°C- Light/Dark	
	Filtered	Unfiltered	Filtered	Unfiltered
0.4 ppt	0.22 ± 0.03	0.71 ± 0.01	0.72 ± 0.00	1.73 ± 0.00
5.4 ppt	0.24 ± 0.05	0.48 ± 0.02	0.68 ± 0.01	1.46 ± 0.00
9.4 ppt	0.22 ± 0.05	0.52 ± 0.00	0.65 ± 0.01	1.27 ± 0.00

Enhanced decay of *E. coli* in filtered water was observed under a light/dark regime compared to dark conditions at 3 and 20°C (Fig. 1), resulting in significant (*p*<0.05) differences in decay rates between light/dark and dark regime (Table 2, Table S8). Remarkably, no significant difference was found between the dark and light/dark regime at 12°C (*p*>0.05) (Table 2, Table S8).

Salinity had no significant (*p*>0.05) effect on the decay of *E. coli* except at 12°C under light/dark regime, where the decay rates of *E. coli* were significantly lower (*p*<0.05) in filtered water with salinities of 5.4 and 9.4 ppt compared to 0.4 ppt (Table 2, Table S10). In summary, the abiotic factors temperature and light/dark regime had a higher influence on the decay of *E. coli* in filtered Rhine River water than the salinity.

The presence of the indigenous microbiota in the Rhine River water microcosms had either a diminishing or enhancing effect on the decay of *E. coli* under the different temperatures and light conditions studied (Fig. 1). Comparing the results in unfiltered to filtered Rhine River water, an enhanced decay of *E. coli* was observed for almost all conditions studied, with *E. coli* incubated at 3°C under a light/dark regime being the exception (Table 2). At this condition, the decay rates were around two times lower in unfiltered water than in filtered water irrespective of the water salinity (Table 2), and which was statistically significant (*p*<0.05, Table S7). Noticeably, the enhanced effect of the indigenous microbiota on decay of *E. coli* was most pronounced under light/dark regime incubated at 20°C (Fig. 1). In addition, the highest decay rate for *E. coli* (1.73 day^−1^) was observed in unfiltered water with a salinity of 0.4 ppt incubated under a light/dark regime at 20°C (Table 2). Furthermore, at 20°C under both the dark and light/dark regime, the decay rate of *E. coli* in unfiltered Rhine River water was significantly (*p*<0.05) lower at a salinity of 5.4 or 9.4ppt than at 0.4ppt (Table 2, Table S10).

The *E. coli* decay caused by the abiotic factors differed in three ways between filtered (i.e., absence of indigenous microbiota) and unfiltered (i.e., presence of indigenous microbiota) Rhine River water. First, the decay of *E. coli* was faster at 20°C than at 3 and 12°C in unfiltered water at all salinities under dark conditions (Fig. 1). The corresponding decay rates at 20°C were significantly (*p*<0.05) higher than at 12°C, whereas such temperature effect was not found in absence of the indigenous microbiota (i.e., filtered water) (Table 2, Table S5). Second, the decay rates in unfiltered water at nearly all salinities and temperatures (excluding 9.4ppt at 3°C) were significantly (*p*<0.05) higher under the light/dark regime than in the dark (Table 2, Table S8). This light/dark versus dark effect is similar, but more pronounced in unfiltered compared to filtered water at 12 and 20°C whereas it is more obvious in filtered than unfiltered at 3°C (Fig. 1, Table 2). Third, a difference in the decay of *E. coli* in unfiltered water with different salinities was only observed at 20°C at dark conditions or under a light/dark regime. This is in contrast to the results from the filtered water samples, where the difference of *E. coli* decay between salinities was only observed at 12°C under a light/dark regime (Fig. 1). In summary, the results from the experiments with unfiltered (i.e., presence of indigenous microbiota) water samples indicated that temperature and light exposure were important abiotic factors affecting the decay rate of *E. coli*, and that the presence of the indigenous microbiota strongly enhanced the decay at temperatures above 12°C.

The interactive effects of the four different factors (salinity, temperature, light, indigenous microbiota) on *E. coli* decay were further statistically quantified by a four-way ANOVA analysis (*p* < 0.05, Table S3). The results from this ANOVA analysis demonstrated that the four tested factors showed interactive influences on *E. coli* decay in Rhine River water. This means that salinity, temperature, light, and presence/absence of the indigenous microbiota influence each other in such a manner that the decay rate is different than what would be expected if looking at only a single environmental condition.

### Decay of *P. aeruginosa* in Rhine River water at different conditions

**Fig. 2:**
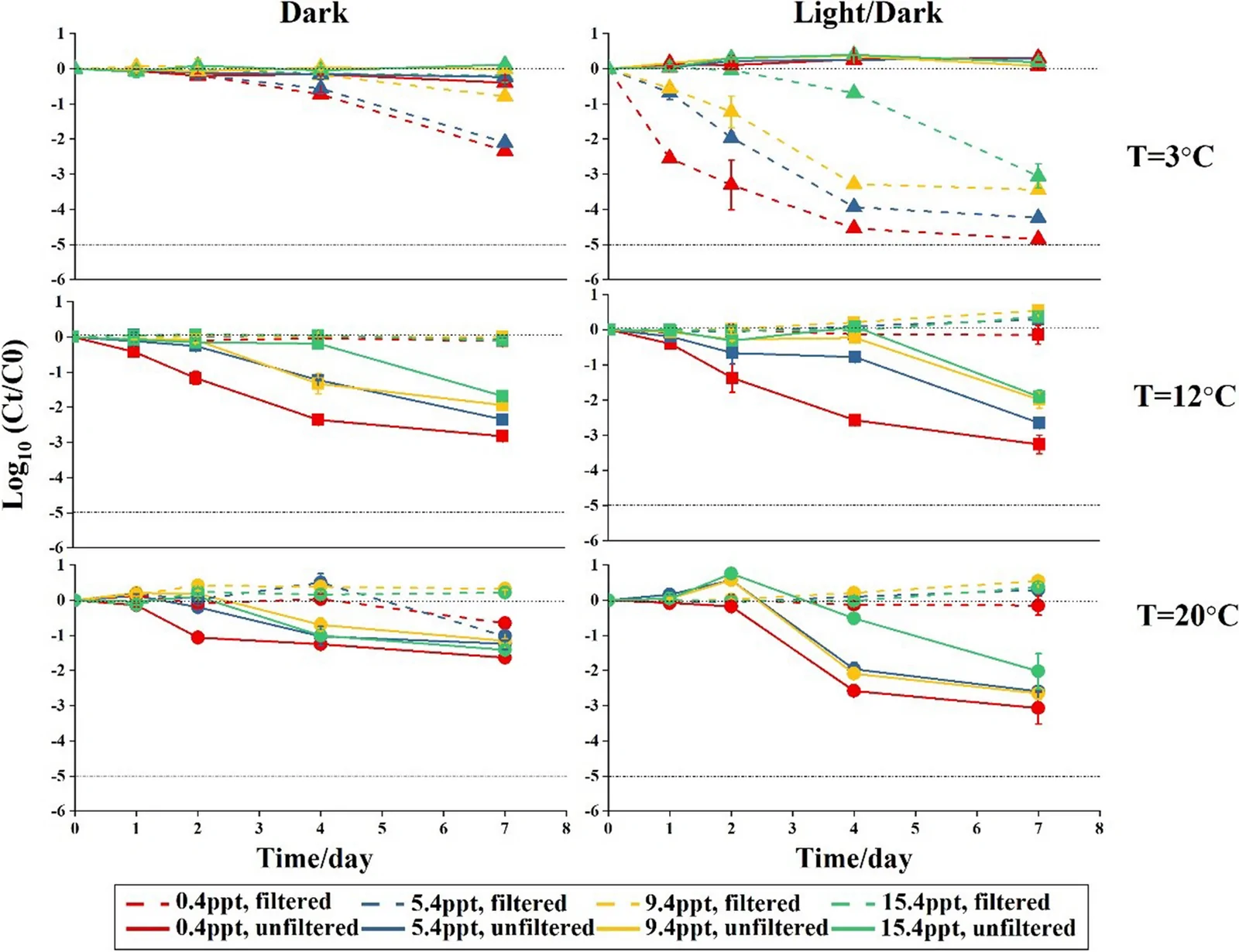
Log_10_ (*C*_*t*_/*C*_0_) of *P. aeruginosa* in filtered and unfiltered Rhine River water that were incubated at various salinity and temperature levels under a dark or a light/dark regime. Data are averages from duplicate bottles (*n*=2); error bars represent standard deviations. The upper dashed line indicates *C*_*t*_=*C*_0_; the bottom dashed line indicates the detection limit in our study

In filtered water under dark conditions, *P. aeruginosa* persists better at higher temperatures (12 and 20°C) than at the lower temperature (3°C) (Fig. 2). An average decay rate of 0.01 day^−1^ was found for all salinity levels at 12°C (Table 3). In contrast, the decay rates for *P. aeruginosa* decreased from 0.28±0.07 to 0.03±0.02 day^−1^ with increasing salinity at 3°C and were significantly higher than decay rates at 12°C with salinities lower than 15.4 ppt (*p*<0.05, Table 3, Table S6, Table S10). This decay trend of *P.aeruginosa* in filtered water caused by temperature remained similar but more obvious under light/dark regime (Fig. 2).

**Table 3:** Log_10_-based decay rates of *P. aeruginosa* in Rhine River water incubated for 7 days under different environmental conditions in microcosms. Data are averages from duplicate bottles (*n*=2); errors (±) represent standard deviations

Log_10_-based decay rate *P. aeruginosa* (day^−1^)				
	3°C—dark		3°C—light/dark	
	Filtered	Unfiltered	Filtered	Unfiltered
0.4ppt	0.28±0.07	0.05±0.01	0.62±0.00	−0.04±0.02
5.4ppt	0.25±0.06	0.03±0.04	0.63±0.00	−0.04±0.01
9.4ppt	0.12±0.01	−0.01±0.01	0.51±0.00	−0.01±0.01
15.4ppt	0.03±0.02	−0.02±0.01	0.44±0.05	−0.03±0.00
	12°C—dark		12°C—light/dark	
	Filtered	Unfiltered	Filtered	Unfiltered
0.4ppt	0.01±0.00	0.42±0.01	0.02±0.03	0.48±0.01
5.4ppt	0.01±0.01	0.35±0.01	−0.05±0.00	0.35±0.01
9.4ppt	0.01±0.00	0.31±0.01	−0.08±0.00	0.26±0.04
15.4ppt	0.02±0.00	0.23±0.01	−0.05±0.00	0.17±0.02
	20°C—dark		20°C—light/dark	
	Filtered	Unfiltered	Filtered	Unfiltered
0.4ppt	0.09±0.02	0.24±0.00	−0.07±0.01	0.51±0.07
5.4ppt	0.13±0.03	0.21±0.04	−0.11±0.01	0.44±0.02
9.4ppt	−0.04±0.00	0.20±0.01	−0.10±0.00	0.44±0.04
15.4ppt	−0.04±0.01	0.23±0.01	−0.14±0.01	0.30±0.09

The influence of light/dark versus dark regime on survival of *P. aeruginosa* in filtered water differed between 3°C and 12/20°C. At 3°C, the decay rates were significantly (*p*<0.05) higher under the light/dark regime compared to the dark regime for all salinity levels (Table S8). However, at 12 and 20°C, decay rates were lower in filtered water under the light/dark regime than under the dark regime, but these differences were statistically significant only in some water salinity levels (*p*<0.05) (Fig. 2, Table 3, Table S8). Furthermore, negative decay rates (suggesting growth of *P. aeruginosa*) were observed in filtered water under the light/dark regime at 20°C with all four salinities and at 12°C with the salinities of 5.4, 9.4, and 15.4 ppt, whereas negative decay rates were only observed under dark conditions at 20°C with salinities of 9.4 and 15.4ppt (Table 3).

**Fig. 3:**
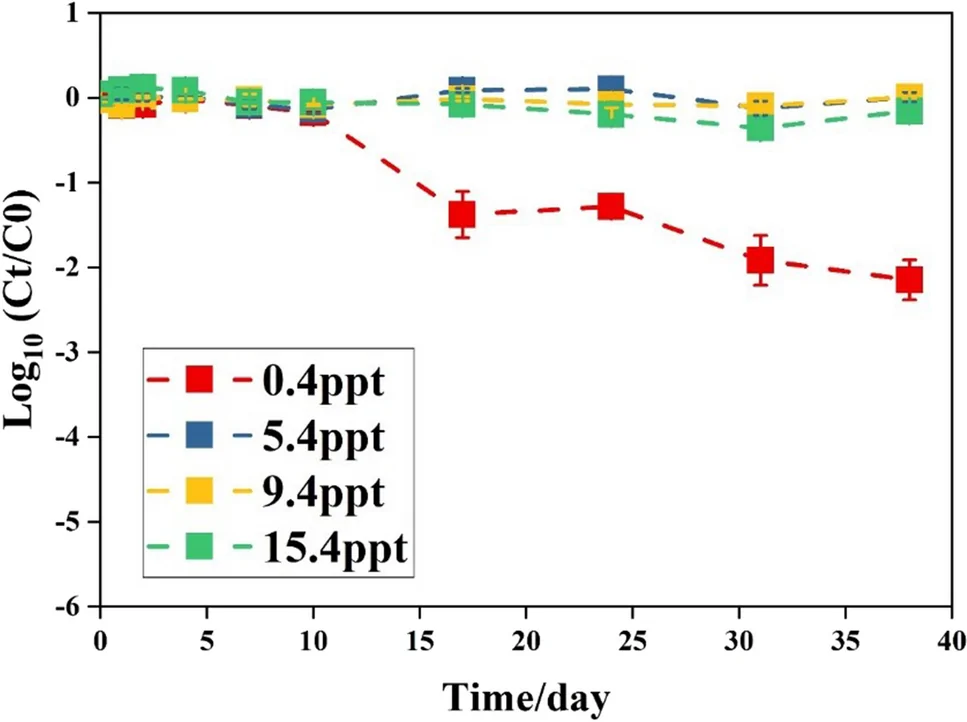
Log_10_ (C_t_/C_0_) of *P. aeruginosa* in filtered river water with various salinity levels at 12°C during 38 days under dark conditions. Data are averages from duplicate bottles (*n*=2); error bars represent standard deviations

Salinity had no clear impact on the decay of *P. aeruginosa* in filtered water under both dark and light/dark regime (Fig. 2). Decay rates of *P. aeruginosa* at salinities above 5.4 ppt were only significantly lower (*p*<0.05) than decay rates of salinities below 5.4 ppt in filtered water under a light/dark regime at 3°C and under dark condition at 3 and 20°C (Table 3). An additional long-term experiment to assess persistence at 12 °C under dark conditions showed that salt concentrations of 5.4, 9.4, and 15.4 ppt resulted in stable *P. aeruginosa* numbers, whereas a salinity of 0.4 ppt resulted in decay of *P. aeruginosa* and the decay rates of *P. aeruginosa* at 0.4 ppt was significantly different from the decay rates at 5.4, 9.4, and 15.4 ppt (*p*<0.01, Fig. 3, Table S10). In summary, as observed for *E. coli*, the abiotic factors temperature and light/dark regime had a higher influence on the decay of *P. aeruginosa* in filtered Rhine River water than the salinity.

The decay of *P. aeruginosa* is also influenced by the presence of indigenous microbiota, but this effect was not consistent over the incubation temperatures applied. The decay of *P. aeruginosa* at 3°C was lower in unfiltered than in filtered water and, correspondingly, the decay rates were significantly (*p*<0.05) lower in unfiltered than in filtered water at 3°C under dark conditions at all salinity levels (Fig. 2, Table 3, Table S7). However, at 12°C and 20°C, the decay of *P. aeruginosa* was higher in unfiltered water compared to filtered water (Fig. 2). Correspondingly, the decay rates of *P. aeruginosa* in unfiltered water under dark conditions with various salinity levels were all significantly (*p*<0.05) higher than in filtered water under the same conditions (Table 3, Table S7).

Next to the observation that decay of *P. aeruginosa* in unfiltered water under dark conditions was higher at 12 and 20°C than at 3°C, decay rates were also significantly (*p*<0.05) higher in unfiltered water at 12°C than at 20°C under dark conditions at all salinity levels (Table 3, Table S6). Under a light/dark regime, *P. aeruginosa* decay at 20°C in unfiltered water was comparable or slightly higher than that at 12°C, which in turn showed a significantly (*p*<0.05) higher decay than at 3°C at all salinities (Table 3, Table S6). An important difference in decay of *P. aeruginosa* in unfiltered water between light/dark and dark regime was found at 20°C, where the decay rates under the light/dark regime were significantly higher (*p*<0.05) than under the dark regime (Fig. 2, Table 3, Table S8). Interestingly, in unfiltered water at 12 and 20 °C, a higher salinity reduced the decay of *P. aeruginosa* compared to lower salinities, which was not observed for filtered water (Fig. 2). This effect is especially obvious at 12°C where, under dark conditions, significant (*p*<0.05) lower decay rates in unfiltered water with salinities above 5.4ppt were observed compared to unfiltered water with a salinity of 0.4ppt (Table 3, Table S10). To summarize, the indigenous microbiota is an important determinant factor on either decay or growth of *P. aeruginosa* in Rhine River water. The abiotic factor temperature is also an important factor that determines *P. aeruginosa* decay in the presence of an indigenous microbiota. Furthermore, the influence of salinity on the decay of *P. aeruginosa* in unfiltered water is more important than the light/dark regime, but less important than temperature.

The interactive effect of the four different environmental conditions on *P. aeruginosa* decay in Rhine River water was also determined with a four-way ANOVA and showed that the four-way interactive effect was not significant (*p*>0.05). However, the three-way interactions between indigenous microbiota, light, and temperature; indigenous microbiota, temperature, and salinity; or light, temperature, and salinity all had a significant interactive effect on *P. aeruginosa* decay in Rhine River water (*p*<0.05, Table S4). However, the three-way interactions between indigenous microbiota, light, and salinity did not have a significant interactive effect on *P. aeruginosa* decay (*p*>0.05).

### *E. coli* versus *P. aeruginosa* decay

A comparison of the decay rates for *E. coli* with the decay rates for *P. aeruginosa* indicates significantly (*p*<0.05) higher decay rates for *E. coli* than *P. aeruginosa* under most environmental conditions (Table S9). A more detailed description on the influence of environmental conditions on the difference in the decay rates between the two bacteria showed that significant (*p*<0.05) higher decay rates for *E. coli* than *P. aeruginosa* were observed for filtered water under most of the different environmental conditions (Tables 2 and 3). The only exception was filtered water incubated at 3°C in the dark where *P. aeruginosa* decayed significantly (*p*<0.05) faster than *E. coli*. At all other conditions in filtered water, the decay rates for *E. coli* were 10 to 33 times higher compared to the decay rates for *P. aeruginosa*. In unfiltered water, the most significant difference in decay rates between *E. coli* and *P. aeruginosa* was observed at 3°C with decay rates of *E. coli* being 12 to 69 times higher than decay rates for *P. aeruginosa*, irrespective of salinity or light. Thus, the fecal indicator bacterium *E. coli* decayed in general much faster in Rhine River water when exposed to combined environmental conditions than the opportunistic pathogen *P. aeruginosa*.

## Discussion

### Key conditions that determine the fate of *E. coli* in surface water

Our study showed that light exposure had a strong effect on the decay rate of *E. coli* in Rhine River water (Table 2, Table 3). Light exposure as a dominant environmental condition on the decay of fecal pathogens in river water has been reported in other studies as well (Korajkic et al. 2014; Bailey et al. 2019; Dean and Mitchell 2022), but these studies only focused on two conditions (light and indigenous microbiota or light and temperature). Nelson et al. (2018) showed that light exposure inactivates *E. coli* by an exogenous mechanism during which a small part of UV-B and visible light are absorbed by chromophores, resulting in the production of photo-produced reactive intermediates which damage and kill the bacterial cells (Nelson et al. 2018). Further studies showed that the light intensity and exposure time are important factors that determine the lethal effect of light exposure on *E. coli*, whereas the suspended solids concentration in the water can diminish the lethal effect of light exposure as a result of light absorbance (Scoullos et al. 2019; Korajkic et al. 2019b).

The presence of indigenous microbiota or higher temperatures enhanced the decay of *E. coli* in Rhine River water as well. These results are consistent with previous observations that also showed prolonged persistence of *E. coli* at lower temperature (4°C or 8°C) in sterilized river water compared to higher temperatures (20, 25, and 37°C) (Wang and Doyle 1998; Ibrahim et al. 2019). In addition, the detrimental effect of the indigenous microbiota on enteric bacteria in water has also been observed before and is important for ecosystem balance (Korajkic et al. 2019b). Successfully competing for nutrients by the indigenous bacteria, exogenous enzyme activity and grazing by protozoa were shown to be influential factors for decay of *E. coli* in river and lake water (Gurijala and Alexander 1990; Bogosian et al. 1996; Jenkins et al. 2011; Wanjugi et al. 2016). For instance, Wanjugi et al. (2016) reported that predation by protozoa contributed 40% and competition 25% to the decay of *E. coli* in surface water (Wanjugi et al. 2016). In addition, we observed no clear influence of salinity on decay of *E. coli*, which was in contrast to Liang et al. (2017) who found that a higher salinity increased the decay of *E. coli* in artificial river water in the absence of indigenous microbiota (Liang et al. 2017). This apparent discrepancy could have been caused by the use of real river water in our study compared to artificial river water in the study of Liang et al. (2017).

An important finding from our study is the interactive effect of the different conditions on *E. coli* decay in Rhine River water. For instance, a significantly higher *E. coli* decay effect of the indigenous microbiota at 3 and 20 °C than at 12°C under dark conditions was observed. At a light/dark regime, however, *E. coli* decay decreased when the indigenous microbiota was present at 3°C. Higher water temperatures have been reported to increase grazer’s activities of the microbiota and this phenomenon has been suggested to be responsible for the increased decay of *E. coli* at higher temperatures compared to lower temperatures (Barcina et al. 1986). Korajkic et al. (2014) showed a dominant influence from ambient sunlight exposure on the decay of *E. coli* in surface water during 120-h experiments (Korajkic et al. 2014). The sunlight exposure, however, also resulted in a lack of observable impact by indigenous microbiota in that study, because the light exposure damaged cells of the indigenous microbiota as well. In contrast, others did not observe a significant interactive effect from sunlight exposure and indigenous microbiota on *E. coli* decay in both marine and freshwater, which might relate to the constant temperature (20.7 °C ± 2.2 in fresh water, 21.0 °C ± 2.0 in marine water) used in that study (Korajkic et al. 2019a). In line with our observations, others also found the effectiveness of insolation with the same light intensities on die-off of the FIB appeared to be seasonal which indicates a light/temperature interaction (Noble et al. 2004; Jenkins et al. 2011). The observation that the effect of an indigenous microbiota was less pronounced at higher salinity could be due to the inactivation of the indigenous predators like protozoa as has been reported by several researchers before (Okabe and Shimazu 2007; Schulz and Childers 2011).

Most studies have only investigated the influence of a single factor or two factors, including the interactive impact, on the decay of *E. coli* (Gurijala and Alexander 1990; Noble et al. 2004; Jenkins et al. 2011; Korajkic et al. 2014; Korajkic et al. 2019a; Barcina et al. 1986). Nevertheless, we showed a significant four-way interactive effect of light, temperature, indigenous microbiota, and salinity on the decay of *E. coli* in Rhine River water, which demonstrates the importance of higher-level interactions than only two environmental conditions. Due to the complex state of four-dimensional effects, it remains difficult to explain the exact nature of the interactive effects between light, temperature, indigenous microbiota, and salinity on the decay of *E. coli* in Rhine River water. Still, it demonstrates the importance of considering combined environmental conditions when studying the decay of *E. coli* in water and predicting its persistence after incidental sewer overflow discharges*.*

### Key conditions that determine the fate of *P. aeruginosa* in surface water

Temperature and the indigenous microbiota had a high impact on the fate of *P. aeruginosa* in Rhine River water in terms of decay or growth in this study. *P. aeruginosa* decayed faster at 3°C compared to 12 and 20°C in the absence of indigenous microbiota, indicating the capability of *P. aeruginosa* to survive at higher temperatures. Previous studies showed that the growth of *P. aeruginosa* depends on the water temperature, and that the temperature range for *P. aeruginosa* growth is broad (from 5 to 42 °C) (Van Asperen et al. 1995; Whitacre 2009; Bomo et al. 2011; Van Der Wielen et al. 2023). Furthermore, it was observed that elevated water temperatures from 15 to 30°C stimulate the growth of *P. aeruginosa* in drinking water biofilms (Van Der Wielen et al. 2023). However, in the present study, it was observed that the indigenous microbiota in Rhine River water was a more important factor responsible for decay of *P. aeruginosa* at 12 and 20°C compared to 3°C. Higher temperatures result in a higher overall microbial activity, including the activity of predators such as protozoa and competing bacteria in water samples (Medema et al. 1997; Ahmed et al. 2021), resulting in higher predation/competition at 12 and 20 than 3°C. However, a possible protection of indigenous microbiota on *P. aeruginosa* at 3°C is difficult to explain.

The presence of light intensified both the decay and growth of *P. aeruginosa* in Rhine River water in this study. As has been mentioned in the “Key conditions that determine the fate of *E. coli* in surface water” section, the light wavelength range investigated in the present study included UV, and UV exposure is a well-known disinfectant method that inactivates bacteria by destroying the DNA in bacterial cells (Nelson et al. 2018). Especially at low temperatures (3°C) in the absence of indigenous microbiota, light had an enhanced effect on the decay of *P. aeruginosa*. However, in the absence of indigenous microbiota but with higher temperatures (12 and 20°C), the light/dark regime enhanced the growth of *P. aeruginosa*.

The results from our study showed a trend of lower decay or even slight growth of *P. aeruginosa* at higher salinities compared to the lowest salinity (0.4ppt) in both the absence and presence of indigenous microbiota. *P. aeruginosa* has been widely isolated from marine water and some of the isolates were regarded as originating from fresh water or sewage (A Mates 1992; Khan et al. 2010; Mohammed et al. 2012; Januário et al. 2019), which demonstrates that *P. aeruginosa* can adapt to survive and possibly grow in water with higher salinities. In addition, Khan et al. (2010) showed the ability of *P. aeruginosa* to grow over a wide range of NaCl concentrations among which the marine strains survived better in higher than in lower salinities (Khan et al. 2010). Moreover, the indigenous microbiota in the Rhine River water might have been partly inactivated at the higher salinities, causing less decay or competition with *P. aeruginosa*, as has been observed for targeted pathogenic bacteria (Schulz and Childers 2011; Liang et al. 2017). This strong survival of *P. aeruginosa* over a wide range of salinity levels is also part of the reason why we tested an additional higher salinity of 15.4ppt to study the persistence of *P. aeruginosa* in urban surface water with higher salinities*.*

In conclusion, a strong interactive effect between the indigenous microbiota and temperature on decay of *P. aeruginosa* was observed and light/dark regime and salinity further intensified the survival trend of *P. aeruginosa*. This is consistent with the ANOVA results that showed several significant three-way interactive effects. As with *E. coli*, these results stress the need to investigate the effect of multiple environmental conditions on waterborne pathogens to reliably predict the fate of these pathogens in water sources.

### Comparison between *E. coli* and *P. aeruginosa* persistence in river Rhine water

The present study shows that *E. coli* decays significantly faster than *P. aeruginosa* under most of the conditions investigated in Rhine River water (Figure S3). These findings are in contrast to a previous study where a significant but weak positive correlation (*R*^2^=0.473) between *E. coli* and *P. aeruginosa* numbers was observed in river water downstream of a wastewater treatment plant effluent (Januário et al. 2019). However, that study only investigated the occurrence of these two bacteria at a specific location in a specific season, which will result in relatively constant environmental conditions. This low variation in environmental condition might explain the apparent discrepancy between their and our study*.* In the present study, water samples were taken at different seasons for the decay experiments with *E. coli* and *P. aeruginosa*, which could have resulted in different indigenous microbiota community structure. Previous studies have demonstrated differences in the bacterial community composition between summer and winter in a river (Kaevska et al. 2016), whereas the protozoan community structure in the water was stable over the seasons (Jiang et al. 2007). The precise influence of the community structure of the indigenous microbiota was, however, beyond the scope of our study. The results from our study showed in general a difference in decay between *E. coli* and *P. aeruginosa*, but under some conditions similar decay were observed for both bacteria (Tables 2 and 3). More importantly, the general faster decay of *E. coli* than *P. aeruginosa* in Rhine River water under different conditions, as observed in our study, indicates that solely relying on FIB, such as *E. coli*, for microbiological water quality monitoring of bathing water could pose a possible public health threat, since opportunistic pathogens as *P. aeruginosa* behave differently than *E. coli* in surface water. Especially, the prolonged persistence in winter and the significantly lower decay in summer of *P. aeruginosa* compared to *E. coli* indicates the relevance of monitoring the opportunistic pathogen *P. aeruginosa* besides FIB in surface water used for recreational activities. Others have also suggested to include *P. aeruginosa* in bathing water quality monitoring and also advocated to implement regulations for non-fecal contamination (Mariño et al. 1994; Mohammed et al. 2012).

Another key finding from our study is that the four investigated conditions (temperature, salinity, light, indigenous microbiota) showed a significant four-way interactive effect on the decay of *E. coli* and three-way interactive effect on decay of *P. aeruginosa*. This finding indicates that *E. coli* and *P. aeruginosa* do not only behave differently to single environmental conditions, but that also the interactive effect of environmental conditions affect *E. coli* and *P. aeruginosa* in a different way. This finding stresses again the importance of applying combined environmental conditions to reliably determine the fate of *E. coli* and *P. aeruginosa* in surface waters.

### The impact of the decay of *E. coli* and *P. aeruginosa* on the quality of swimming water in Amsterdam

The variation in light/dark conditions, salinity (0.4, 5.4, and 9.4ppt), temperature (3, 12, and 20 °C), and absence/presence of indigenous microbiota in the present study was based on the conditions that can be observed in the urban surface water system in Amsterdam throughout the year (Van Der Meulen et al. 2023). As such, the results of this study can be used to predict the behavior of these two bacteria during periods of low (winter) and high swimming activity (summer) in Amsterdam. Both *E. coli* and *P. aeruginosa* showed the highest persistence in winter (with indigenous microbiota, 3°C, and light/dark) and the fastest decay in summer (with indigenous microbiota, 20°C, and light/dark). This indicates a higher risk of exposure to these pathogens for swimmers when swimming activities are low. The actual decay of *E. coli* and *P. aeruginosa* in Rhine River water in the summer could even be higher than observed in our study. Both the sunlight intensity and exposure time are higher in the summer period in the Netherlands compared to the light intensity applied in the present study. Other studies have shown that higher light intensity in summer significantly increases the decay of *E. coli* (Scoullos et al. 2019). Although the microbial risk for swimming/recreation in the urban water system of Amsterdam might be lower in summer compared to winter, a considerable risk might still occur at specific moments in the summer. Heavy rainfalls in the summer, which occur more frequently due to climate change, cause sewage overflow into surface waters, resulting in relative high numbers of waterborne pathogens, as was the case in the Amsterdam City Swim in 2015 mentioned in the “Introduction” section (Hintaran et al. 2018). The lower decay rate for *P. aeruginosa* compared to *E. coli* under typical summer conditions indicates that only measuring *E. coli* as an indicator microorganisms for pathogen contamination after such rainfall events is insufficient, since it underestimates the presence and decay of opportunistic pathogens, such as *P. aeruginosa*.

Another aspect in the Amsterdam urban water system is that the salinity of surface water is expected to increase due to saltwater intrusion and longer periods of drought (Delsman et al. 2018). Therefore, since the present study shows that higher salinities aid the persistence of both *E. coli* and *P. aeruginosa*, the risk of health issues as a result of coming in contact with both fecal and opportunistic pathogens through recreational activities in Amsterdam surface waters might increase in the future. Furthermore, it is important to stress that the surface water quality differs throughout the city’s waterways in Amsterdam. For instance, the salinity can differ between 1 and 9 ppt depending on the location in the Amsterdam urban water system (Zoutgehalte - Rijkswaterstaat Waterinfo 2023). This could result in a longer persistence of *E. coli* and *P. aeruginosa* in the water at distinct location in the city which demonstrates the importance of a widespread monitoring network for the microbial water quality at locations commonly used for recreational activities, such as swimming.

## Supplementary Information

ESM 1 (PDF 220 kb)

## Data Availability

The datasets generated during and/or analyzed during the current study are available from the corresponding author on reasonable request.

## References

[CR1] Ahmed W, Toze S, Veal C, Fisher P, Zhang Q, Zhu Z, Staley C, Sadowsky MJ (2021) Comparative decay of culturable faecal indicator bacteria, microbial source tracking marker genes, and enteric pathogens in laboratory microcosms that mimic a sub-tropical environment. Sci Total Environ 751:141475. 10.1016/j.scitotenv.2020.141475 32890804 10.1016/j.scitotenv.2020.141475

[CR2] Atlas RM, Bartha R (1998) Microbial ecology: fundamentals and applications. Microb Evol Biodivers

[CR3] Aw T (2019) Environmental aspects and features of critical pathogen groups. In: Michigan State University, Rose JB, Jiménez Cisneros B, UNESCO - International Hydrological Programme (eds) Water and Sanitation for the 21st Century: Health and Microbiological Aspects of Excreta and Wastewater Management (Global Water Pathogen Project). Michigan State University

[CR4] Bailey ES, Casanova LM, Sobsey MD (2019) Effects of environmental storage conditions on survival of indicator organisms in a blend of surface water and dual disinfected reclaimed water. J Appl Microbiol 126:985–994 30592123 10.1111/jam.14186

[CR5] Barcina I, Arana I, Iriberri J, Egea L (1986) Influence of light and natural microbiota of the Butrón river on *E. coli* survival. Antonie van Leeuwenhoek 52(6):555–566. 10.1007/BF00423416 3545073 10.1007/BF00423416

[CR6] Bogosian G, Sammons LE, Morris PJ, O’Neil JP, Heitkamp MA, Weber DB (1996) Death of the *Escherichia coli K-12* strain *W3110* in soil and water. Appl Environ Microbiol 62:4114–4120. 10.1128/aem.62.11.4114-4120.1996 8900002 10.1128/aem.62.11.4114-4120.1996 PMC168233

[CR7] Bomo A-M, Tryland I, Haande S, Hagman C, Utkilen H (2011) The impact of *cyanobacteria* on growth and death of opportunistic pathogenic bacteria. Water Sci Technol J Int Assoc Water Pollut Res 64:384–390. 10.2166/wst.2011.647 10.2166/wst.2011.647 22097011

[CR8] Chick H (1908) An investigation of the laws of disinfection. J Hyg (Lond) 8:92–158. 10.1017/S0022172400006987 20474353 10.1017/s0022172400006987 PMC2167134

[CR9] Collier SA, Deng L, Adam EA, Benedict KM, Beshearse EM, Blackstock AJ, Bruce BB, Derado G, Edens C, Fullerton KE, Gargano JW, Geissler AL, Hall AJ, Havelaar AH, Hill VR, Hoekstra RM, Reddy SC, Scallan E, Stokes EK et al (2021) Estimate of burden and direct healthcare cost of infectious waterborne disease in the United States. Emerg Infect Dis 27:140–149. 10.3201/eid2701.190676 33350905 10.3201/eid2701.190676 PMC7774540

[CR10] Dean K, Mitchell J (2022) Identifying water quality and environmental factors that influence indicator and pathogen decay in natural surface waters. Water Res 211:118051 35051677 10.1016/j.watres.2022.118051

[CR11] Delsman JR, Van Baaren ES, Siemon B, Dabekaussen W, Karaoulis MC, Pauw PS, Vermaas T, Bootsma H, De Louw PGB, Gunnink JL, Dubelaar CW, Menkovic A, Steuer A, Meyer U, Revil A, Oude Essink GHP (2018) Large-scale, probabilistic salinity mapping using airborne electromagnetics for groundwater management in Zeeland, the Netherlands. Environ Res Lett 13:084011. 10.1088/1748-9326/aad19e

[CR12] Directive EU (2006) 7/EC of the European Parliament and of the Council of 15 February 2006 concerning the management of bathing water quality and repealing Directive 76/160/EEC. Off J Eur Union 2013:L64

[CR13] Gurijala KR, Alexander M (1990) Explanation for the decline of bacteria introduced into lake water. Microb Ecol 20:231–244. 10.1007/BF02543879 24193976 10.1007/BF02543879

[CR14] He Y, Sutton NB, Rijnaarts HHH, Langenhoff AAM (2016) Degradation of pharmaceuticals in wastewater using immobilized TiO_2_ photocatalysis under simulated solar irradiation. Appl Catal B Environ 182:132–141. 10.1016/j.apcatb.2015.09.015

[CR15] Hintaran AD, Kliffen SJ, Lodder W, Pijnacker R, Brandwagt D, van der Bij AK, Siedenburg E, Sonder GJB, Fanoy EB, Joosten RE (2018) Infection risks of city canal swimming events in the Netherlands in 2016. PLOS ONE 13:e0200616. 10.1371/journal.pone.0200616 30052633 10.1371/journal.pone.0200616 PMC6063404

[CR16] Ibrahim EME, El-Liethy MA, Abia ALK, Hemdan BA, Shaheen MN (2019) Survival of *E. coli O157: H7*, *Salmonella Typhimurium*, *HAdV2* and *MNV-1* in river water under dark conditions and varying storage temperatures. Sci Total Environ 648:1297–1304 30340275 10.1016/j.scitotenv.2018.08.275

[CR17] Januário AP, Afonso CN, Mendes S, Rodrigues MJ (2019) Faecal indicator bacteria and *Pseudomonas aeruginosa* in Marine Coastal Waters: is there a relationship? Pathogens 9:13. 10.3390/pathogens9010013 31877730 10.3390/pathogens9010013 PMC7169392

[CR18] Jenkins MB, Fisher DS, Endale DM, Adams P (2011) Comparative die-off of *Escherichia coli 0157: H7* and fecal indicator bacteria in pond water. Environ Sci Technol 45:1853–1858 21306148 10.1021/es1032019

[CR19] Jiang J-G, Wu S-G, Shen Y-F (2007) Effects of seasonal succession and water pollution on the protozoan community structure in an eutrophic lake. Chemosphere 66:523–532. 10.1016/j.chemosphere.2006.05.042 16822536 10.1016/j.chemosphere.2006.05.042

[CR20] Kaevska M, Videnska P, Sedlar K, Slana I (2016) Seasonal changes in microbial community composition in river water studied using 454-pyrosequencing. SpringerPlus 5:409. 10.1186/s40064-016-2043-6 27069829 10.1186/s40064-016-2043-6 PMC4821842

[CR21] Khan NH, Ahsan M, Taylor WD, Kogure K (2010) Culturability and survival of marine, freshwater and clinical *Pseudomonas aeruginosa*. Microbes Environ 25:266–274 21576881 10.1264/jsme2.me09178

[CR22] Korajkic A, McMinn BR, Ashbolt NJ, Sivaganesan M, Harwood VJ, Shanks OC (2019a) Extended persistence of general and cattle-associated fecal indicators in marine and freshwater environment. Sci Total Environ 650:1292–1302. 10.1016/j.scitotenv.2018.09.108 30308816 10.1016/j.scitotenv.2018.09.108 PMC8982556

[CR23] Korajkic A, McMinn BR, Harwood VJ (2018) Relationships between microbial indicators and pathogens in recreational water settings. Int J Environ Res Public Health 15:2842 30551597 10.3390/ijerph15122842 PMC6313479

[CR24] Korajkic A, McMinn BR, Shanks OC, Sivaganesan M, Fout GS, Ashbolt NJ (2014) Biotic interactions and sunlight affect persistence of fecal indicator bacteria and microbial source tracking genetic markers in the Upper Mississippi River. Appl Environ Microbiol 80:3952–3961. 10.1128/AEM.00388-14 24747902 10.1128/AEM.00388-14 PMC4054226

[CR25] Korajkic A, Wanjugi P, Brooks L, Cao Y, Harwood VJ (2019b) Persistence and decay of fecal microbiota in aquatic habitats. Microbiol Mol Biol Rev 83:e00005–e00019. 10.1128/MMBR.00005-19 31578217 10.1128/MMBR.00005-19 PMC7405076

[CR26] Korajkic A, Wanjugi P, Harwood VJ (2013) Indigenous microbiota and habitat influence *Escherichia coli* survival more than sunlight in simulated aquatic environments. Appl Environ Microbiol 79:5329–5337 23811514 10.1128/AEM.01362-13 PMC3753954

[CR27] Liang L, Goh SG, Gin KYH (2017) Decay kinetics of microbial source tracking (MST) markers and human *adenovirus* under the effects of sunlight and salinity. Sci Total Environ 574:165–175. 10.1016/j.scitotenv.2016.09.031 27631197 10.1016/j.scitotenv.2016.09.031

[CR28] Mariño FJ, Moriñigo MA, Martinez-Manzanares E (1994) Borrego JJ (1995) Microbiological-epidemiological study of selected marine beaches in Malaga (Spain). Health-Relat Water Microbiol 31:5–9. 10.1016/0273-1223(95)00232-C

[CR29] Mates A (1992) The significance of testing for *Pseudomonas aeruginosa* in recreational seawater beaches. Microbios 71:89–93 1453986

[CR30] Medema GJ, Bahar M, Schets FM (1997) Survival of *Cryptosporidium parvum*, *Escherichia coli*, faecal *enterococci* and *Clostridium* perfringens in river water: influence of temperature and autochthonous microorganisms. Water Sci Technol 35:249–252

[CR31] Mena KD, Gerba CP (2009) Risk assessment of *Pseudomonas aeruginosa* in water. In: Whitacre DM (ed) Reviews of Environmental Contamination and Toxicology, vol 201. Springer, US, Boston, MA, pp 71–115 10.1007/978-1-4419-0032-6_3 19484589

[CR32] Menon P, Billen G, Servais P (2003) Mortality rates of autochthonous and fecal bacteria in natural aquatic ecosystems. Water Res 37:4151–4158 12946897 10.1016/S0043-1354(03)00349-X

[CR33] Mohammed RL, Echeverry A, Stinson CM, Green M, Bonilla TD, Hartz A, McCorquodale DS, Rogerson A, Esiobu N (2012) Survival trends of *Staphylococcus aureus*, *Pseudomonas aeruginosa*, and *Clostridium* perfringens in a sandy South Florida beach. Mar Pollut Bull 64:1201–1209. 10.1016/j.marpolbul.2012.03.010 22516512 10.1016/j.marpolbul.2012.03.010

[CR34] Nelson KL, Boehm AB, Davies-Colley RJ, Dodd MC, Kohn T, Linden KG, Liu Y, Maraccini PA, McNeill K, Mitch WA (2018) Sunlight-mediated inactivation of health-relevant microorganisms in water: a review of mechanisms and modeling approaches. Environ Sci Process Impacts 20:1089–1122 30047962 10.1039/c8em00047f PMC7064263

[CR35] Noble RT, Lee IM, Schiff KC (2004) Inactivation of indicator micro-organisms from various sources of faecal contamination in seawater and freshwater. J Appl Microbiol 96:464–472 14962126 10.1111/j.1365-2672.2004.02155.x

[CR36] Okabe S, Shimazu Y (2007) Persistence of host-specific *Bacteroides–Prevotella* 16S rRNA genetic markers in environmental waters: effects of temperature and salinity. Appl Microbiol Biotechnol 76:935–944. 10.1007/s00253-007-1048-z 17598108 10.1007/s00253-007-1048-z

[CR37] Peters S, Ouboter M, Lugt KVD, Koop S, Leeuwen KV (2021) Retrospective analysis of water management in Amsterdam, The Netherlands. Water 13:1099. 10.3390/w13081099

[CR38] Rodríguez-Zaragoza S (1994) Ecology of free-living amoebae. Crit Rev Microbiol 20:225–241 7802958 10.3109/10408419409114556

[CR39] Saha P, Wagner TV, Ni J, Langenhoff AAM, Bruning H, Rijnaarts HHM (2020) Cooling tower water treatment using a combination of electrochemical oxidation and constructed wetlands. Process Saf Environ Prot 144:42–51. 10.1016/j.psep.2020.07.019

[CR40] Sales-Ortells H, Agostini G, Medema G (2015) Quantification of waterborne pathogens and associated health risks in urban water. Environ Sci Technol 49:6943–6952. 10.1021/acs.est.5b00625 25932966 10.1021/acs.est.5b00625

[CR41] Schets FM, Schijven JF, de Roda Husman AM (2011) Exposure assessment for swimmers in bathing waters and swimming pools. Water Res 45:2392–2400 21371734 10.1016/j.watres.2011.01.025

[CR42] Schets FM, Van Den Berg HHJL, Lynch G, De Rijk S, De Roda Husman AM, Schijven JF (2020) Evaluation of water quality guidelines for public swimming ponds. Environ Int 137:105516. 10.1016/j.envint.2020.105516 32007691 10.1016/j.envint.2020.105516

[CR43] Schets FM, Van Wijnen JH, Schijven JF, Schoon H, De Roda Husman AM (2008) Monitoring of waterborne pathogens in surface waters in Amsterdam, The Netherlands, and the potential health risk associated with exposure to *Cryptosporidium* and *Giardia* in these waters. Appl Environ Microbiol 74:2069–2078. 10.1128/AEM.01609-07 18281429 10.1128/AEM.01609-07 PMC2292590

[CR44] Schulz CJ, Childers GW (2011) Fecal *Bacteroidales* diversity and decay in response to variations in temperature and salinity. Appl Environ Microbiol 77:2563–2572. 10.1128/AEM.01473-10 21278280 10.1128/AEM.01473-10 PMC3126366

[CR45] Scoullos IM, Lopez Vazquez CM, van de Vossenberg J, Hammond M, Brdjanovic D (2019) Effect of artificial solar radiation on the die-off of pathogen indicator organisms in urban floods. Int J Environ Res 13:107–116 30873212 10.1007/s41742-018-0160-5 PMC6383957

[CR46] Sinton LW, Davies-Colley RJ, Bell RG (1994) Inactivation of *enterococci* and fecal coliforms from sewage and meat works effluents in seawater chambers. Appl Environ Microbiol 60:2040–2048. 10.1128/aem.60.6.2040-2048.1994 8031097 10.1128/aem.60.6.2040-2048.1994 PMC201599

[CR47] Sokolova E, Åström J, Pettersson TJR, Bergstedt O, Hermansson M (2012) Decay of *Bacteroidales* genetic markers in relation to traditional fecal indicators for water quality modeling of drinking water sources. Environ Sci Technol 46:892–900. 10.1021/es2024498 22148545 10.1021/es2024498

[CR48] U.S. Environmental Protection Agency (2014) Method 1603: *Escherichia coli* (*E. coli*) in water by membrane filtration using modified membrane-thermotolerant Escherichia coli Agar (Modified MTEC); EPA-821-R-14-010

[CR49] Van Asperen IA, De Rover CM, Schijven JF, Oetomo SB, Schellekens JFP, Van Leeuwen NJ, Colle C, Havelaar AH, Kromhout D, Sprenger MWJ (1995) Risk of otitis externa after swimming in recreational fresh water lakes containing *Pseudomonas aeruginosa*. BMJ 311:1407–1410. 10.1136/bmj.311.7017.1407 8520277 10.1136/bmj.311.7017.1407 PMC2544405

[CR50] Van Der Meulen ES, Van De Ven FHM, Van Oel PR, Rijnaarts HHM, Sutton NB (2023) Improving suitability of urban canals and canalized rivers for transportation, thermal energy extraction and recreation in two European delta cities. Ambio 52:195–209. 10.1007/s13280-022-01759-3 36001251 10.1007/s13280-022-01759-3 PMC9666579

[CR51] Van Der Wielen PWJJ, Dignum M, Donocik A, Prest EI (2023) Influence of temperature on growth of four different opportunistic pathogens in drinking water biofilms. Microorganisms 11:1574. 10.3390/microorganisms11061574 37375076 10.3390/microorganisms11061574 PMC10303289

[CR52] Wagner TV, Helmus R, Becker E, Rijnaarts HHM, De Voogt P, Langenhoff AAM, Parsons JR (2020) Impact of transformation, photodegradation and interaction with glutaraldehyde on the acute toxicity of the biocide DBNPA in cooling tower water. Environ Sci Water Res Technol 6:1058–1068. 10.1039/C9EW01018A

[CR53] Wang G, Doyle MP (1998) Survival of Enterohemorrhagic *Escherichia coli O157:H7* in Water. J Food Prot 61:662–667. 10.4315/0362-028X-61.6.662 9709245 10.4315/0362-028x-61.6.662

[CR54] Wanjugi P, Fox GA, Harwood VJ (2016) The interplay between predation, competition, and nutrient levels influences the survival of *Escherichia coli* in aquatic environments. Microb Ecol 72:526–537. 10.1007/s00248-016-0825-6 27484343 10.1007/s00248-016-0825-6

[CR55] Whitacre DM (ed) (2009) Reviews of Environmental Contamination and Toxicology, vol 201. Springer, US, Boston, MA

[CR56] Whitman RL, Nevers MB, Korinek GC, Byappanahalli MN (2004) Solar and temporal effects on *Escherichia coli* concentration at a Lake Michigan Swimming Beach. Appl Environ Microbiol 70:4276–4285. 10.1128/AEM.70.7.4276-4285.2004 15240311 10.1128/AEM.70.7.4276-4285.2004 PMC444827

[CR57] Wnlfe RL (1990) Ultraviolet disinfixtion of potable water. Environ Sci Technol. 24:768–773. 10.1021/es00076a001

[CR58] World Health Organization (2003) Guidelines for safe recreational water environments: Coastal and fresh waters. 34351725

[CR59] Zoutgehalte - Rijkswaterstaat Waterinfo. https://waterinfo.rws.nl/#/publiek/zouten. Accessed 17 Sep 2023

