# Supplementary material for: Influence of combined abiotic/biotic factors on decay of P. aeruginosa and E. coli in Rhine River water
Source: Appl Microbiol Biotechnol. 2024 Apr 10;108(1):294. doi: 10.1007/s00253-024-13128-z (PMC11399167; doi:10.1007/s00253-024-13128-z)
Supplement: Supplementary file 1 — (PDF 220 kb) [file 253_2024_13128_MOESM1_ESM.pdf]

Applied Microbiology and Biotechnology

Influence of combined abiotic/biotic factors on decay of *P. aeruginosa* and *E. coli* in Rhine River water

Sha Gao[1], Nora B. Sutton[*,1], Thomas V. Wagner[1], Huub H. M. Rijnaarts[1], Paul W. J. J. van der Wielen [2,3]

[1] Department of Environmental Technology, Wageningen University, PO Box 17, 6700 EV Wageningen, The Netherlands

[2] KWR Water Research Institute, Groningenhaven 7, 3433PE Nieuwegein, the Netherlands

[3] Laboratory of Microbiology, Wageningen University, PO Box 17, 6700 EV Wageningen, The Netherlands

*Corresponding Author: nora.sutton@wur.nl; tel. +31 (0)317 483339

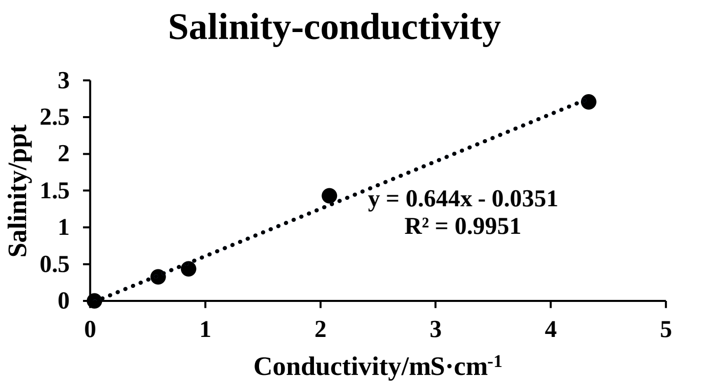

Figure S1 Standard curve of Salinity and Conductivity at 20°C by a digital conductivity sensor (HACH HQ440d, *Germany)*

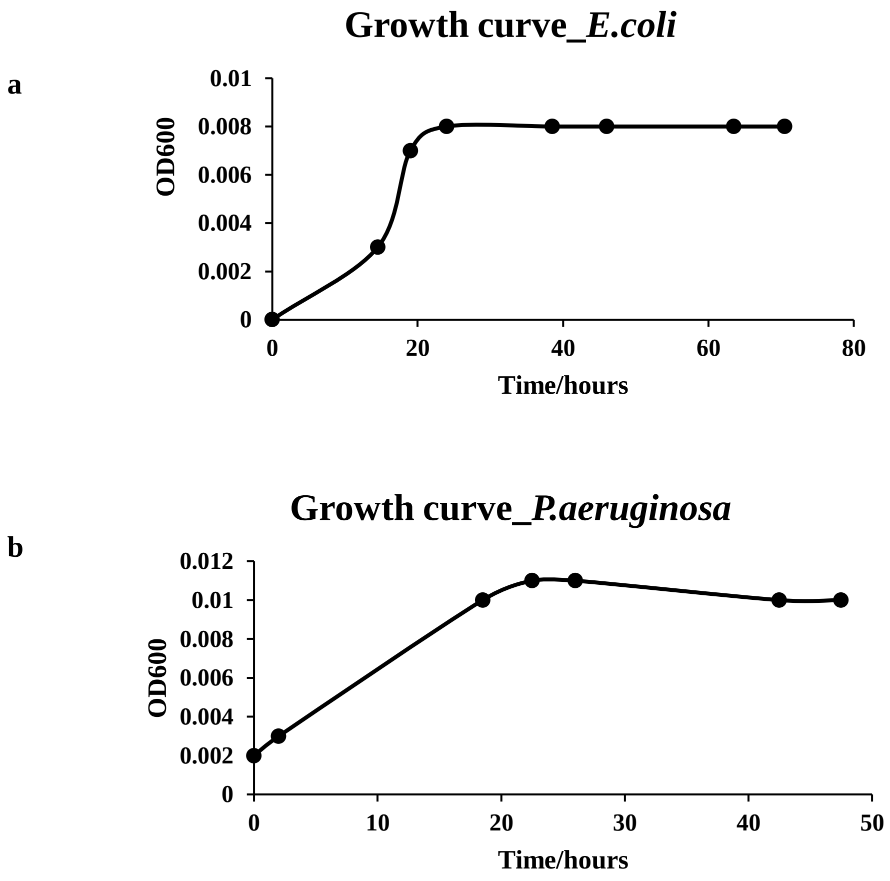

Figure S2 Growth curve of **a.** *E. coli*, and **b.** *P. aeruginosa* in M9 with 10mg/L Glucose.

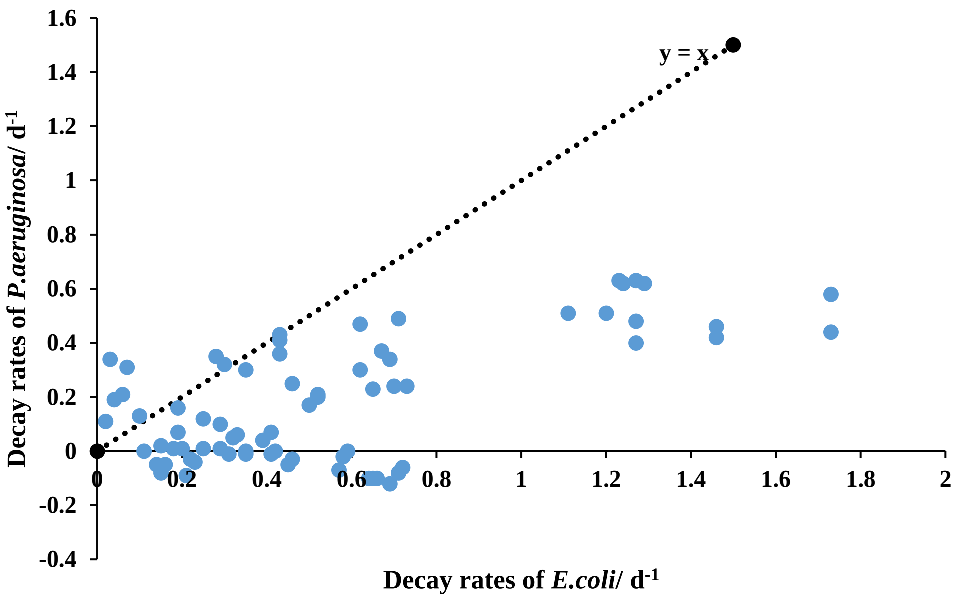

Figure S3 Scatter plot comparison for decay rates of *E. coli* and *P. aeruginosa* under all given conditions

Table S1 Physiochemical parameters of Rhine River water samples during the experiment of *E. coli* under all conditions

Conditions			pH	Conductivity (µS·cm^{-1})	Salinity (ppt)	Water temperature (℃)	NH_4^+ (mg·L^{-1})	COD (mg·L^{-1})
3℃	Light/dark	F&UF	8.16±0.12	847±250	0.51±0.16	8.5	0.01±0.00	13.80±0.02
	Dark	F&UF	8.01±001	715±2	0.43±0.00	8.3	0.11±0.00	6.26±0.32
12℃	Light/dark	F&UF	8.32±0.05	775±88	0.46±0.06	8.9	0.10±0.00	22.50±1.20
	Dark	F&UF	8.18±0.05	785±37	0.47±0.02	12.9	0.08±0.01	9.00±0.11
20℃	Light/dark	F&UF	8.22±0.01	558±2	0.32±0.00	8.5	0.11±0.01	21.65±0.35
	Dark	F&UF	8.21±0.06	786±24	0.47±0.02	12.9	0.10±0.00	9.89±0.03

Abbreviations: F: Filtered; UF: Unfiltered.

Table S2 Physiochemical parameters of Rhine River water samples during the experiment of *P. aeruginosa* under all conditions

Conditions			pH	Conductivity (µS·cm^{-1})	Salinity (ppt)	Water temperature (℃)	NH_4^+ (mg·L^{-1})	COD (mg·L^{-1})
12℃	Dark	UF	7.85±0.14	633±3	0.37±0.00	19.6	0.22±0.00	16.20±0.30
		F	8.13±0.02	632±12	0.37±0.01	19.6	0.01±0.01	12.57±3.04
	Light/dark	UF	8.36±0.03	647±0	0.38±0.00	21	0.01±0.00	10.15±0.05
		F	8.29±0.05	628±2	0.37±0.00	21	0.01±0.00	12.75±0.35
3℃	Dark	UF&F	7.84±0.04	747±5.5	0.45±0.00	21.5	0.05±0.00	15.05±0.35
	Light/dark	UF&F	7.71±0.01	675±3	0.40±0.01	21.5	0.18±0.01	13.25±0.45
20℃	Dark	UF&F	8.1	529	0.31	16.5	N.A.	N.A.
	Light/dark	UF&F	8.2	525	0.3	16.5	N.A.	N.A.

Abbreviations: F: Filtered; UF: Unfiltered.

Table S3 Summary of four factor variance ANOVA analysis (factors: indigenous microbiota, light exposure, temperature, salinity) evaluating the interactive impacts on *E.coli* by using the decay rates. Statistically significant values are displayed in bold (α = 0.05).

**Tests of Between-Subjects Effects**					
**Source**	Type III Sum of Squares	df	Mean Square	F	Sig.
Corrected Model	12.444[a]	35	0.356	201.416	<.001
Intercept	21.572	1	21.572	12219.89	<.001
Indigenous	0.95	1	0.95	538.103	<.001
Light	4.435	1	4.435	2512.485	<.001
Temperature	1.742	2	0.871	493.448	<.001
Salinity	0.132	2	0.066	37.521	<.001
**Indigenous * Light**	0.045	1	0.045	25.209	**<.001**
**Indigenous * Temperature**	1.843	2	0.922	522.074	**<.001**
**Indigenous * Salinity**	0.025	2	0.013	7.219	**0.002**
**Light * Temperature**	1.057	2	0.528	299.314	**<.001**
**Light * Salinity**	0.026	2	0.013	7.422	**0.002**
**Temperature * Salinity**	0.049	4	0.012	6.935	**<.001**
**Indigenous * Light * Temperature**	1.997	2	0.999	565.74	**<.001**
Indigenous * Light * Salinity	0	2	0	0.062	0.94
**Indigenous * Temperature * Salinity**	0.089	4	0.022	12.638	**<.001**
Light * Temperature * Salinity	0.019	4	0.005	2.621	0.051
**Indigenous * Light * Temperature * Salinity**	0.034	4	0.009	4.849	**0.003**
Error	0.064	36	0.002		
Total	34.08	72			
Corrected Total	12.508	71			
a. R Squared = .995 (Adjusted R Squared = .990)					

Table S4 Summary of four factor variance ANOVA analysis (factors: indigenous microbiota, light exposure, temperature, salinity) evaluating the interactive impacts on *P. aeruginosa* by using the decay rates. Statistically significant values displayed are in bold (α = 0.05).

**Tests of Between-Subjects Effects**					
Source	Type III Sum of Squares	df	Mean Square	F	Sig.
Corrected Model	4.259[a]	47	0.091	61.737	<.001
Intercept	2.404	1	2.404	1637.588	<.001
indigenous	0.283	1	0.283	192.648	<.001
light	0.072	1	0.072	49.091	<.001
temperature	0.016	2	0.008	5.607	0.006
salinity	0.207	3	0.069	46.905	<.001
indigenous * light	0.001	1	0.001	0.869	0.356
**indigenous * temperature**	2.714	2	1.357	924.454	**<.001**
indigenous * salinity	0.008	3	0.003	1.812	0.158
**light * temperature**	0.173	2	0.086	58.785	**<.001**
light * salinity	0.006	3	0.002	1.331	0.275
temperature * salinity	0.01	6	0.002	1.119	0.366
**indegenious * light * temperature**	0.606	2	0.303	206.481	**<.001**
indigenous * light * salinity	0.011	3	0.004	2.522	0.069
**indegenious * temperature * salinity**	0.102	6	0.017	11.588	**<.001**
**light * temperature * salinity**	0.031	6	0.005	3.562	**0.005**
indegenious * light * temperature * salinity	0.019	6	0.003	2.175	0.062

Error	0.07	48	0.001
Total	6.733	96	
Corrected Total	4.329	95	
a. R Squared = .984 (Adjusted R Squared = .968)			

Table S5 The p values from one way ANOVA test with a Bonferroni post hoc test for statistic difference of decay rates of *E.coli* between temperatures

**One way ANOVA with a Bonferroni post hoc test**				
	EDF_Sig.	ELF_Sig.	EDUF_Sig.	ELUF_Sig.
3 vs 12℃	0.000319476	5.80128E-09	1.000	0.204707059
3 vs 20℃	4.25503E-05	6.18114E-09	0.002	4.26934E-09
12 vs 20℃	0.871106001	9.20233E-09	0.001	3.99705E-08

*. The mean difference is significant at the 0.05 level.
Abbreviations: E**:** *E.coli*; F: Filtered; UF: Unfiltered; L: Light/dark; D: Dark.

Table S6 The p values from Kruskal-Wallis H test with a Bonferroni post hoc test for statistic difference of decay rates of *P.aeruginosa* between temperatures

**Kruskal-Wallis H test with a Bonferroni post hoc test**				
	FDP_Adj. Sig.[a]	FLP_Adj. Sig.[a]	UFDP_Adj. Sig.[a]	UFLP_Adj. Sig.[a]
3 vs 12℃	0.044	0.053	0.000	0.013
3 vs 20℃	0.059	2.92964E-05	0.036	0.000260792
12 vs 20 ℃	1.000	0.121	0.231	0.867

a. significance values have been adjusted by the Bonferroni correction for multiple tests.
Abbreviations: P: *P.aeruginosa*; F: Filtered; UF: Unfiltered; L: Light/dark; D: Dark.

Table S7 The p values from one way ANOVA test for statistic difference of decay rates of *E.coli* and *P.aeruginosa* in filtered (without indigenous microbiota) and unfiltered (with indigenous microbiota) water

**Filtered vs Unfiltered (one way ANOVA)**			
	Sig.		Sig.
T3DE	1.12024E-08	T3DP	0.003807
T12DE	0.0013	T12DP	1.0857E-08
T20DE	0.000034	T20DP	0.000038
T3LE	8.3763E-09	T3LP	4.047E-11
T12LE	2.3276E-07	T12LP	0.000003
T20LE	0.000003	T20LP	1.4387E-09

Abbreviations: T3: 3℃; T12: 12℃; T20: 20℃; E: *E.coli*; P: *P.aeruginosa*; L: Light/dark; D: Dark.

Table S8 The p values from one way ANOVA test for statistic difference of decay rates of *E.coli* and *P.aeruginosa* in light/dark and dark conditions

**Light/dark vs Dark (one way ANOVA)**			
	Sig.		Sig.
T3FE	1.3478E-12	T3FP	0.000004
T12FE	0.66636	T12FP	0.005241
T20FE	7.5149E-10	T20FP	0.000445
T3UFE	0.00306	T3UFP	0.00692
T12UFE	0.000003	T12UFP	0.801509
T20UFE	0.000003	T20UFP	0.000109

Abbreviations: T3: 3℃; T12: 12℃; T20: 20℃; E: *E.coli*; P: *P.aeruginosa*; F: Filtered; UF: Unfiltered;

Table S9 The p values from student t-test for statistic difference of decay rates between *E.coli* and *P.aeruginosa* in all given conditions

**Student t-test**	
E.coli vs *P.aeruginosa*	P(T<=t) two-tail<br>2.58128E-14

Table S10 The p values from one way ANOVA test with a Bonferroni post hoc test for statistic difference of decay rates of *E.coli* and *P.aeruginosa* between different salinity levels

**One way ANOVA with a Bonferroni post hoc test**							
Salinities	T3UFDE_Sig.	T3FDE_Sig.	T3UFLE_Sig.	T3FLE_Sig.	T12UFDE_Sig.	T12FDE_Sig.	
0.4vs5.4ppt	0.343178275	1	0.608145324	1	0.99126	0.287799091	
0.4vs9.4ppt	1	1	1	0.324769463	0.60083	1	
5.4vs9.4ppt	0.207319337	1	1	0.448409469	1	0.51949541	
	T12UFLE_Sig.	T12FLE_Sig.	T20UFDE_Sig.	T20FDE_Sig.	T20UFLE_Sig.	T20FLE_Sig.	
0.4vs5.4ppt	1	0.019501332	0.004527372	1	--	0.169455844	
0.4vs9.4ppt	1	0.035490225	0.007493246	1	--	0.040587036	
5.4vs9.4ppt	1	0.852429897	0.522488049	1	--	0.341047149	
	T3UFDP_Sig.	T3FDP_Sig	T3UFLP_Sig	T3FLP_Sig	T12UFDP_Sig	T12FDP_Sig	
0.4vs5.4ppt	1	1	1	1	0.00776	1	
0.4vs9.4ppt	0.656877405	0.433903335	0.810923708	0.1819511	0.00109	1	
0.4 vs15.4ppt	0.411555821	0.108119184	1	0.041465611	0.00013	1	
5.4vs9.4ppt	1	0.713998348	0.805926293	0.132628727	0.03629	1	
5.4 vs15.4ppt	0.859000736	0.160513649	1	0.032789059	0.00069	1	
9.4vs15.4 ppt	1	1	1	0.848661088	0.00382	1	
	T12UFLP_Sig	T12FLP_Sig	T20UFDP_Sig	T20FDP_Sig	T20UFLP_Sig	T20FLP_Sig	T12FDP_38days_Sig
0.4vs5.4ppt	0.076102558	0.21790249	1	1	1	0.17278007	0.002
0.4vs9.4ppt	0.010004826	0.05966464	1	0.069239459	1	0.444445752	0.002
0.4vs15.4ppt	0.002573146	0.205232327	1	0.071361797	0.40807	0.035084223	0.004
5.4vs9.4ppt	0.191577736	1	1	0.028462075	1	1	1.000
5.4vs15.4ppt	0.018096649	1	1	0.029178342	1	0.682327045	0.939
9.4vs15.4 ppt	0.19853047	1	1	1	1	0.250262517	1.000

Abbreviations: T3: 3℃; T12: 12℃; T20: 20℃; E: *E.coli*; P: *P.aeruginosa*; F: Filtered; UF: Unfiltered; D: Dark; L: Light/dark
